# Lesbian, gay, bisexual, and/or transgender (LGBT) cultural competency across the intersectionalities of gender identity, sexual orientation, and race among healthcare professionals

**DOI:** 10.1371/journal.pone.0277682

**Published:** 2022-11-11

**Authors:** Dustin Z. Nowaskie, Sidrah Najam

**Affiliations:** 1 Department of Psychiatry and Behavioral Sciences, Keck School of Medicine of University of Southern California, Los Angeles, California, United States of America; 2 Department of Psychiatry, Indiana University School of Medicine, Indianapolis, Indiana, United States of America; Stanford University School of Medicine, UNITED STATES

## Abstract

**Background:**

There is some data regarding lesbian, gay, bisexual, and transgender (LGBT) cultural competency among healthcare professionals. While few studies have indicated differences in competency between heterosexual and sexual minority professionals, no known studies have assessed LGBT cultural competency among diverse groups with multiple minority identities. This study aimed to characterize healthcare professionals’ LGBT cultural competency by comparing twelve different demographically diverse healthcare professional groups based on gender identity, sexual orientation, and race.

**Methods:**

Deidentified data (N = 2254) was aggregated from three independent studies (i.e., healthcare professional students, psychiatry residents, and dementia care providers). A series of multivariate analyses of covariance were conducted with groups (based on gender identity, sexual orientation, and race), other demographic variables as independent variables, and LGBT-Development of Clinical Skills Scale scores (Overall LGBT-DOCSS, Clinical Preparedness, Attitudinal Awareness, and Basic Knowledge) as dependent variables.

**Findings:**

Compared to men, women reported significantly higher LGBT-DOCSS scores, except significantly lower Clinical Preparedness. Compared to cisgender, heterosexual professionals, cisgender, sexual minority professionals and gender minority professionals reported significantly higher LGBT-DOCSS scores. There were several other differences among groups, such as heterosexual, cisgender, White/Caucasian men reporting low LGBT-DOCSS scores but high Clinical Preparedness; heterosexual, cisgender, White/Caucasian women with high LGBT-DOCSS scores except Clinical Preparedness; heterosexual, racial minority professionals with low LGBT-DOCSS scores; and gender, sexual, and racial minority professionals with the highest LGBT-DOCSS scores.

**Conclusions:**

There are subtle, yet important, differences in LGBT cultural competency among healthcare professionals. More diversity, intersectionality, and multiple minority identities appear to lead to higher competency. Appreciating these gender, sexual, and racial minority professionals’ unique perspectives may promote the development of better, more culturally affirming LGBT health education.

## Introduction

Cultural competency has become an increasingly important conceptual model within healthcare settings that helps dictate patient-provider interactions. As the United States becomes increasingly diverse, known racial and ethnic gaps in access to preventative care have shown that the current healthcare model falls behind in meeting patient needs [1]. Disparities in access to equitable healthcare are often associated with worse health outcomes, and it is likely that these outcomes are at least partially perpetuated by a lack of cultural competency among providers and students (this group of individuals is, henceforth, collectively referred to as “healthcare professionals”, unless specified otherwise). Furthermore, improved cultural competency is often associated with better healthcare outcomes and experiences [1]. In addition to deficits in cultural competency, biases among healthcare professionals may play a role in the delivery of care [2], and some data exist to address biases among physicians in treating patients who identify as minorities. Hall et al. [3] noted implicit biases among healthcare professionals in regards to race and an interaction between parameters such as age, gender identity, and sexual orientation [3, 4]. However, research on implicit biases within healthcare settings have tended to focus on race and less so on other identifying characteristics [3]. Among these biases, both sexual orientation and gender identity are associated with stigma and discrimination, but seldomly have been studied in the context of the current healthcare system.

“Minority” (this term, henceforth, collectively represents gender, sexual, and/or racial minorities, unless otherwise stated) healthcare professionals have been seldomly surveyed with a paucity of studies evaluating this group of individuals’ cultural competency. Also, to our knowledge, there is no present national demographic data concerning sexual minority and gender minority healthcare professionals. Even if such data existed, it would likely be grossly underrepresented, as 30–60% of sexual minority and gender minority medical students conceal their identities, partially secondary to fear of discrimination [5]. One study on sexual minority and gender minority students navigating medical school showed that non-White sexual minority and gender minority medical students experienced their education both with sexual identity stigma and racial discrimination, which increased stress and impacted their ability to build strong faculty mentorships [6].

The consequences of deficient cultural competency have numerous implications, given as many as 40% of lesbian, gay, bisexual, and/or transgender (LGBT) patients experience some degree of discrimination in healthcare settings [7]. In regards to the LGBT patient population, healthcare professionals hold biases, infrequently address gender identity and sexual orientation in patient-care settings, and show limitations in education and cultural competency [8–10]. Furthermore, differences in cultural competency exist among healthcare professionals, with some studies showing demographic variables to be significant predictors of LGBT cultural competency [8, 9]. These variables, which include gender identity, sexual orientation, and race, lend to the notion that there are subtle differences between diverse healthcare professionals in their LGBT cultural competency. However, despite many demographic variables being known significant predictors of LGBT cultural competency, studies have not directly compared groups based on gender identity, sexual orientation, and race [8–10]. Likewise, there are no known studies evaluating LGBT cultural competency among demographically diverse healthcare professional groups. Given the discrimination faced by the LGBT patient population, the role of healthcare professionals in addressing the disproportionate poor health outcomes and suicidality among LGBT people is crucial.

Healthcare is a continually evolving field, with increasing diversity and intersectionality among healthcare professionals within a backdrop of cultural shifts in how differences are perceived. Meyer coined the minority stress model, which suggests that overt stigma and discrimination toward LGBT communities may lead to internalized stress and eventually poor health outcomes and suicidality [11]. Minority stressors are important when considering the overlap of multiple identities, a concept known as intersectionality. Rooted in Black feminist thought, intersectionality suggests that many people belong to various social categories, some of which confer privileges or disadvantages [12]. Individuals who represent multiple minority identities may consequently have multiple minority stressors, which may lead to additional social and healthcare challenges [12]. Previous studies have also studied intersectionality within the context of heteronormativity, reporting findings that cisgender, heterosexual people of color reported that they felt privileged by their heterosexuality but were also able to identify and empathize with the LGBT population based on their own experiences with discrimination [12]. Contrastingly, Tan and colleagues have found that those who are ethnic minorities and carry additional minority identities may not necessarily experience greater minority stress due to increased resiliency [13]. As intersectionality in essence is premised on the concept of overlapping prejudice and how it shapes living in the world, an intriguing inquiry is whether those healthcare professionals who are a part of communities with multiple minority identities (and likely experience minority stress themselves) have differing LGBT cultural competency. As such, the goals of this study were to characterize the LGBT cultural competency of healthcare professionals with multiple minority identities by 1) comparing LGBT cultural competency between cisgender, heterosexual healthcare professionals, sexual minority healthcare professionals, and gender minority healthcare professionals; 2) comparing LGBT cultural competency between Caucasian and non-Caucasian healthcare professionals; and 3) comparing twelve different demographically diverse healthcare professional groups based on gender identity, sexual orientation, and race. Overall, we hypothesized that healthcare professionals with multiple minority identities would report significantly higher LGBT cultural competency.

## Methods

### Study design and variables

This study was considered not human subjects research by the Indiana University Institutional Review Board (Protocol #10799). Deidentified data was aggregated from recent three independent studies that evaluated healthcare professionals’ LGBT cultural competency with the same study methodology but with entirely different healthcare professional samples, including healthcare professional students [9], psychiatry residents [8], and dementia care providers [10]. In each of those studies, demographics (i.e., age, gender identity, sexual orientation, race, ethnicity, type of healthcare discipline, and region), experiential variables (i.e., total hours of LGBT education received), and the LGBT-Development of Clinical Skills Scale (LGBT-DOCSS) [14] were collected, with the exception that total LGBT hours was not collected in the provider study [10]. Information pertaining to specific demographics, experiential variables, and LGBT-DOCSS scores of each pooled sample can be found in respective publications [8–10].

The LGBT-DOCSS is a self-reported clinical assessment. All LGBT-DOCSS items consist of 7-point Likert scales (1 = strongly disagree, 4 = somewhat agree/disagree, 7 = strongly agree). There is an overall mean score (“Overall LGBT-DOCSS”) as well as three subscales (“Clinical Preparedness”, “Attitudinal Awareness”, and “Basic Knowledge”). Higher scores indicate more perceived clinical preparedness and knowledge and less prejudice regarding LGBT patients.

### Statistical methods

Results were analyzed using SPSS Statistics 26 (IBM Corp., Armonk, NY). Demographic frequencies and LGBT-DOCSS score means were computed. To compare groups of interest, participants were collapsed based on gender identity, sexual orientation, and/or race—comparison #1: women healthcare professionals (i.e., both cisgender women and transgender women), men healthcare professionals (i.e., both cisgender men and transgender men), and gender diverse healthcare professionals (who did not identify exclusively as binary transgender, i.e., nonbinary and other gender identity healthcare professionals); comparison #2: heterosexual healthcare professionals and sexual minority healthcare professionals (i.e., lesbian, gay, bisexual, queer, and other sexual identity professionals); comparison #3: cisgender, heterosexual healthcare professionals; cisgender, sexual minority healthcare professionals; and gender minority healthcare professionals (including binary transgender healthcare professionals, nonbinary healthcare professionals, and other gender identity healthcare professionals); comparison #4: White/Caucasian healthcare professionals, Asian/Asian American healthcare professionals, Black/African American healthcare professionals, and other racial identity healthcare professionals (including multiracial healthcare professionals); and comparison #5: twelve groups differing by gender identity (cisgender men healthcare professionals, cisgender women healthcare professionals, and gender minority healthcare professionals), sexual orientation (heterosexual healthcare professionals and sexual minority healthcare professionals), and race (White/Caucasian healthcare professionals and racial minority healthcare professionals). A series of multivariate analyses of covariance (MANCOVAs) were conducted with the groups of interest, other demographic variables (i.e., age, ethnicity, type of healthcare discipline, and region) as independent variables and covariates, and LGBT-DOCSS scores as dependent variables. Significant differences between the groups of interest were examined further by post-hoc Fisher’s Least Significant Differences tests. While statistical comparisons were done with professionals who identified with “other” or combinations of terms, their differences were not expounded on in order to avoid generalizations of these heterogeneous groups. All MANCOVAs and post-hoc tests were repeated by including total LGBT hours (i.e., both curricular and extracurricular education) as an additional independent variable. Given the number of independent and dependent variables, consequent multiplicity of tests, and thus increased likelihood of making a type I error, statistical significance was set at *a* = 0.001.

## Results

A total of 2254 healthcare professionals were analyzed. Healthcare professionals were diverse in terms of age (range 18–69 years old), gender identity (29.0% cisgender men, 68.5% cisgender women, and 2.5% gender minorities), sexual orientation (82.4% heterosexual, 1.5% lesbian, 3.8% gay, 7.6% bisexual, 2.2% queer, and 2.5% identifying with other or combinations of terms), races (71.6% White/Caucasian, 16.0% Asian/Asian American, 4.3% Black/African American, and 8.2% identifying with other or combinations of terms), ethnicity (92.9% not Hispanic and/or Latino and 7.1% Hispanic and/or Latino), discipline (81.9% healthcare professional students and 18.1% providers, majority with Doctor of Medicine and Doctor of Osteopathic Medicine degrees), and region (3.2% Northeast, 72.1% Midwest, 5.6% South, and 18.8% West).

**Fig 1:**
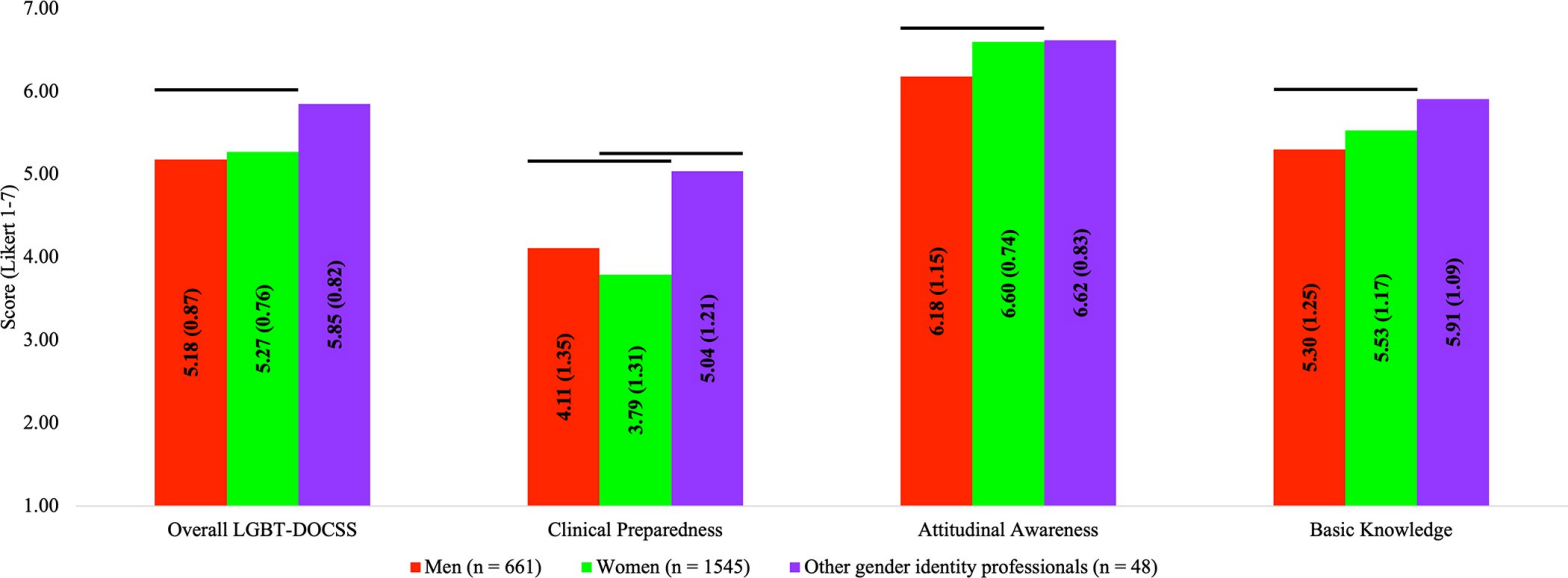
LGBT cultural competency across gender identity. Abbreviations: DOCSS, Development of Clinical Skills Scale; LGBT, Lesbian, Gay, Bisexual, and Transgender. Bars represent significant comparisons p < 0.001. Healthcare professionals were categorized by gender identity, and there were significant group differences on LGBT-DOCSS scores.

In comparison #1, there were significant group differences on LGBT-DOCSS scores: Overall LGBT-DOCSS [*F*(2,2226) = 11.051, *p* < 0.001], Clinical Preparedness [*F*(2,2226) = 11.230, *p* < 0.001], Attitudinal Awareness [*F*(2,2226) = 66.429, *p* < 0.001], and Basic Knowledge [*F*(2,2226) = 11.735, *p* < 0.001]. Women healthcare professionals reported significantly higher LGBT-DOCSS scores, except Clinical Preparedness, which was significantly lower, than men healthcare professionals (Fig 1). Compared to women healthcare professionals, other gender identity healthcare professionals reported significantly higher Clinical Preparedness.

**Fig 2:**
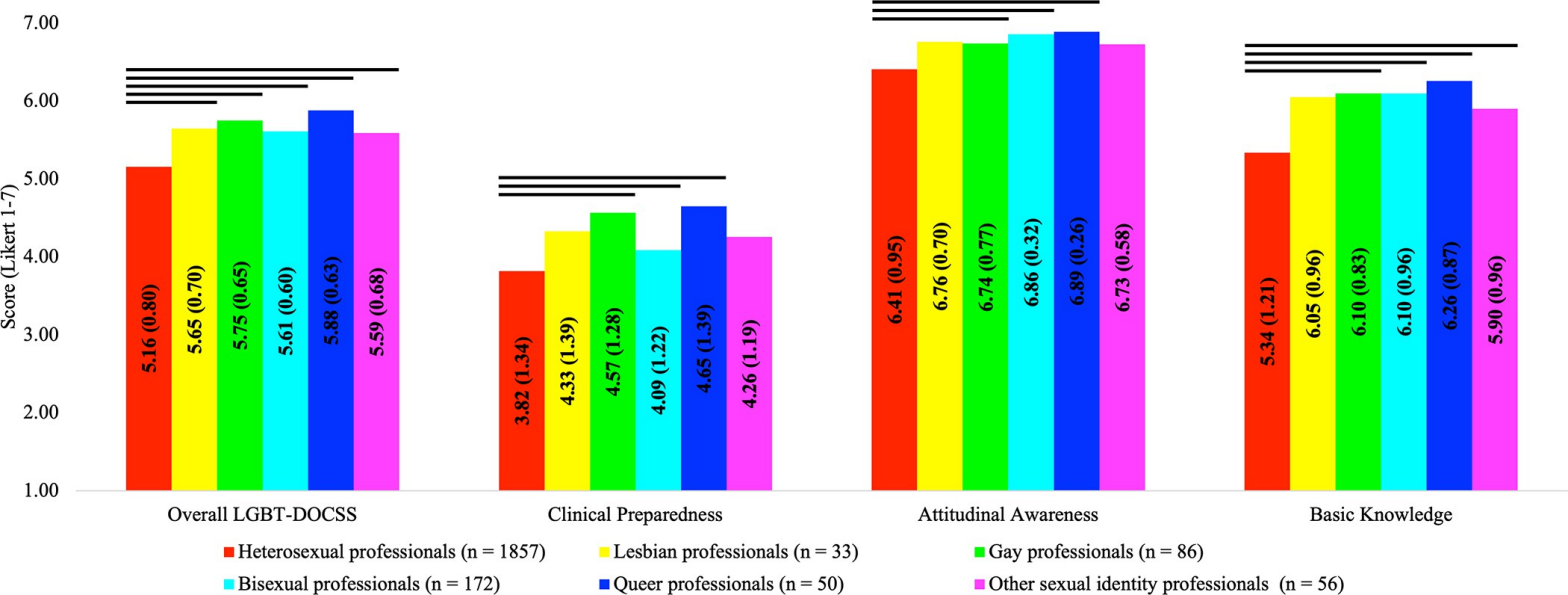
LGBT cultural competency across sexual orientation. Abbreviations: DOCSS, Development of Clinical Skills Scale; LGBT, Lesbian, Gay, Bisexual, and Transgender. Bars represent significant comparisons p < 0.001. Healthcare professionals were categorized by sexual orientation, and there were significant group differences on LGBT-DOCSS scores.

In comparison #2, there were significant group differences on LGBT-DOCSS scores: Overall LGBT-DOCSS [*F*(5,2226) = 29.645, *p* < 0.001], Clinical Preparedness [*F*(5,2226) = 8.622, *p* < 0.001], Attitudinal Awareness [*F*(5,2226) = 17.535, *p* < 0.001], and Basic Knowledge [*F*(5,2226) = 25.333, *p* < 0.001]. Compared to heterosexual healthcare professionals, healthcare professionals who identified as gay, bisexual, and queer reported significantly higher LGBT-DOCSS scores (Fig 2). While lesbian healthcare professionals reported significantly higher Overall LGBT-DOCSS than heterosexual healthcare professionals, their other LGBT-DOCSS scores were not significantly higher. Other sexual identity healthcare professionals reported significantly higher Overall LGBT-DOCSS and Basic Knowledge than heterosexual healthcare professionals. There were no differences in LGBT-DOCSS scores between sexual minority professionals, i.e., there were no differences across lesbian, gay, bisexual, queer, and other sexual identity healthcare professionals.

**Fig 3:**
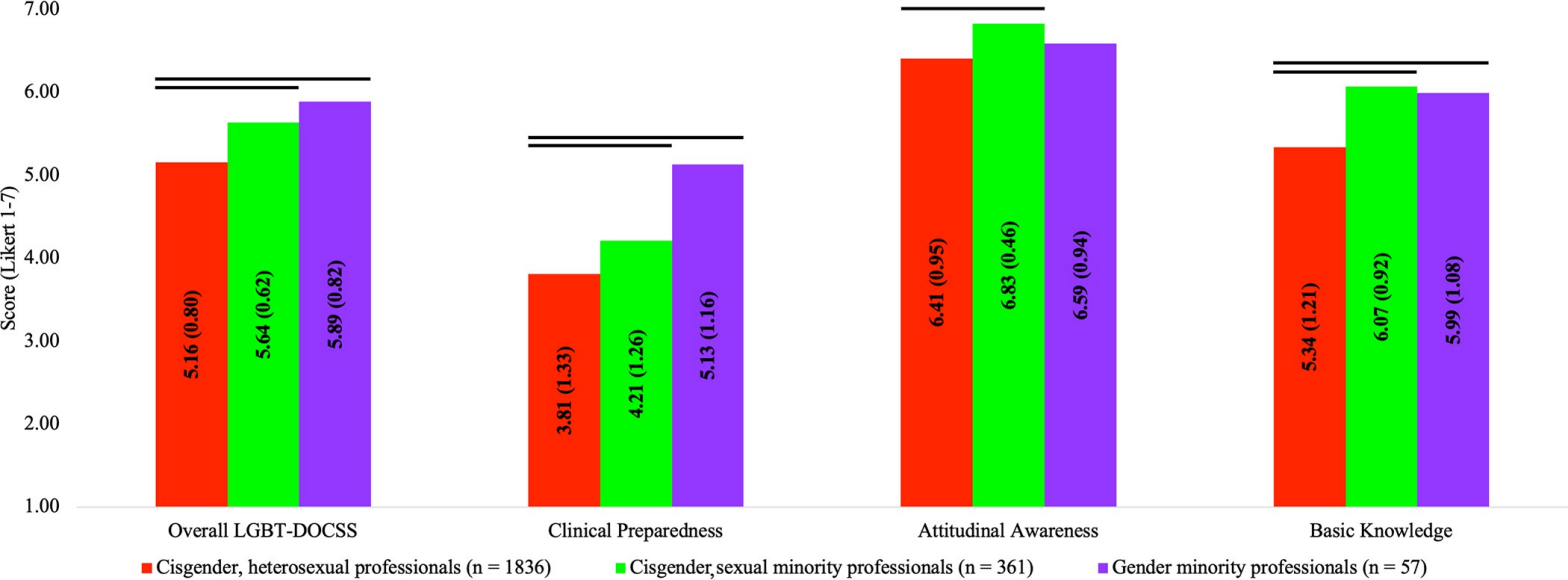
LGBT cultural competency across sexual orientation and gender identity. Abbreviations: DOCSS, Development of Clinical Skills Scale; LGBT, Lesbian, Gay, Bisexual, and Transgender. Bars represent significant comparisons p < 0.001. Healthcare professionals were categorized by sexual orientation and gender identity, and there were significant group differences on LGBT-DOCSS scores.

In comparison #3, there were significant group differences on LGBT-DOCSS scores: Overall LGBT-DOCSS [*F*(2,2231) = 74.385, *p* < 0.001], Clinical Preparedness [*F*(2,2231) = 30.521, *p* < 0.001], Attitudinal Awareness [*F*(2,2231) = 34.284, *p* < 0.001], and Basic Knowledge [*F*(2,2231) = 61.483, *p* < 0.001]. Cisgender, sexual minority healthcare professionals and gender minority healthcare professionals reported significantly higher LGBT-DOCSS scores than cisgender, heterosexual healthcare professionals, with the exception that there were no significant differences in Attitudinal Awareness between gender minority healthcare professionals and cisgender, heterosexual healthcare professionals (Fig 3).

**Fig 4:**
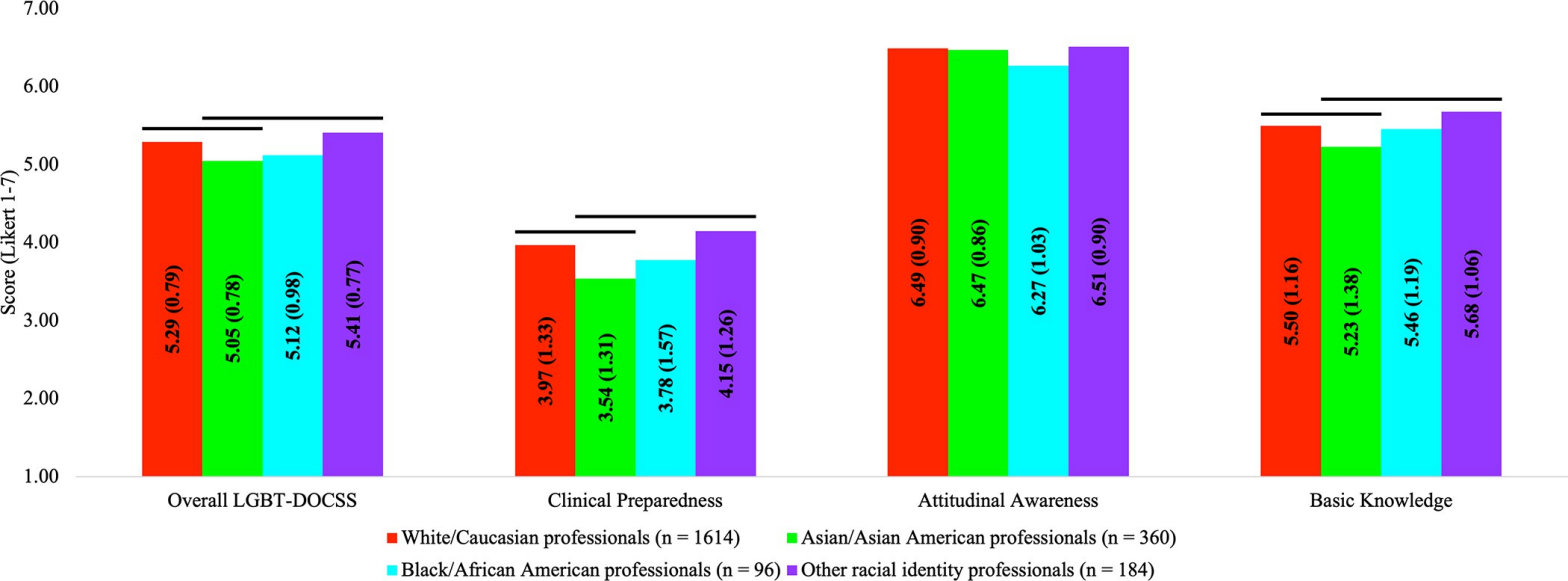
LGBT cultural competency across race. Abbreviations: DOCSS, Development of Clinical Skills Scale; LGBT, Lesbian, Gay, Bisexual, and Transgender. Bars represent significant comparisons p < 0.001. Healthcare professionals were categorized by race, and there were significant group differences on LGBT-DOCSS scores.

In comparison #4, there were significant group differences on LGBT-DOCSS scores: Overall LGBT-DOCSS [*F*(3,2226) = 11.035, *p* < 0.001], Clinical Preparedness [*F*(3,2226) = 14.391, *p* < 0.001], and Basic Knowledge [*F*(3,2226) = 5.474, *p* < 0.001]. Compared to Asian/Asian American healthcare professionals, White/Caucasian healthcare professionals and other racial identity healthcare professionals reported significantly higher LGBT-DOCSS scores, except Attitudinal Awareness (Fig 4).

**Fig 5:**
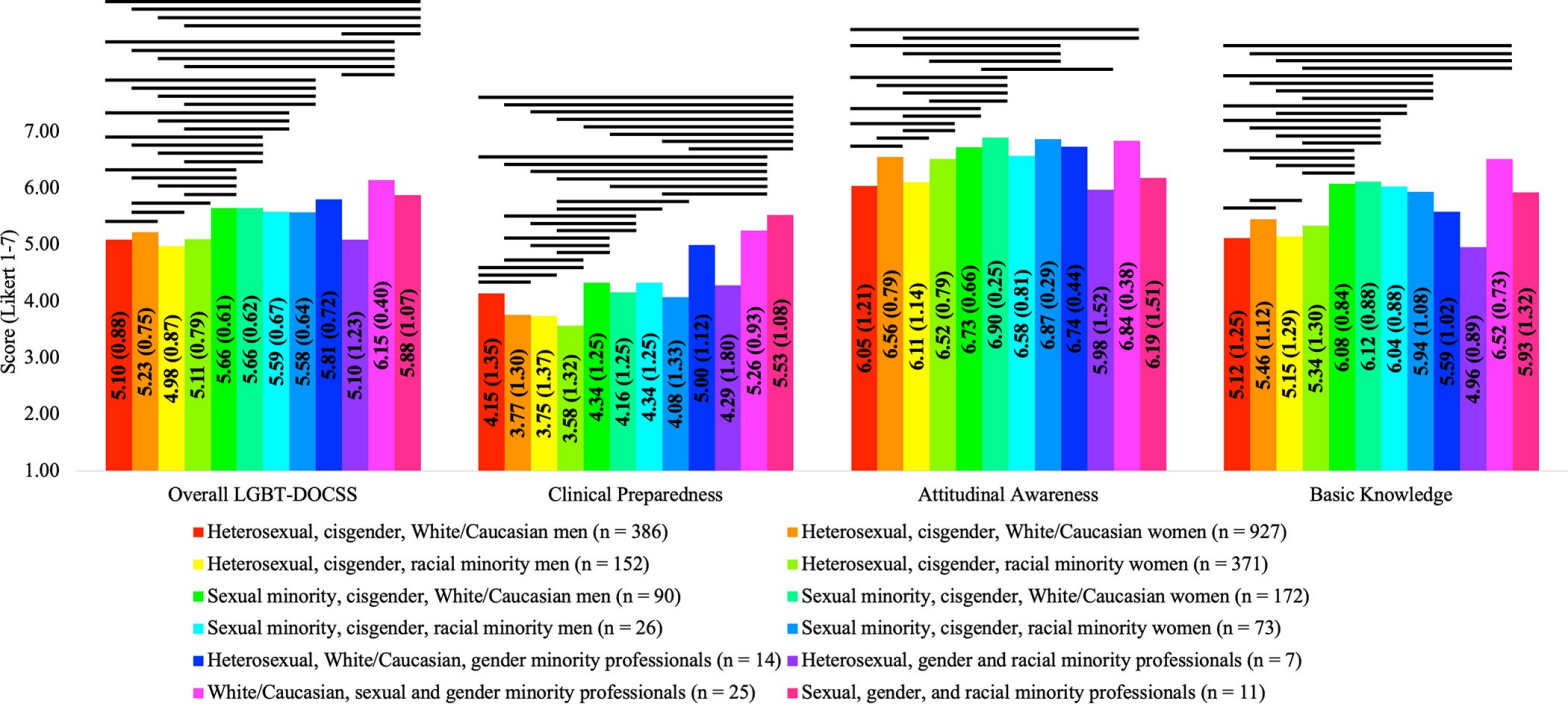
LGBT cultural competency across gender identity, sexual orientation, and race. Abbreviations: DOCSS, Development of Clinical Skills Scale; LGBT, Lesbian, Gay, Bisexual, and Transgender. Bars represent significant comparisons p < 0.001. Healthcare professionals were categorized by gender identity, sexual orientation, and race, and there were significant group differences on LGBT-DOCSS scores.

In comparison #5, there were significant group differences on LGBT-DOCSS scores: Overall LGBT-DOCSS [*F*(11,2225) = 19.136, *p* < 0.001], Clinical Preparedness [*F*(11,2225) = 11.015, *p* < 0.001], Attitudinal Awareness [*F*(11,2225) = 19.214, *p* < 0.001], and Basic Knowledge [*F*(11,2225) = 15.447, *p* < 0.001]. Generally, healthcare professionals who identified with multiple minority identities reported significantly higher LGBT-DOCSS scores (Fig 5). Compared to heterosexual, cisgender men (both White/Caucasian and racial minority) healthcare professionals, healthcare professionals who identified as heterosexual, cisgender, White/Caucasian women reported higher LGBT-DOCSS scores, except Clinical Preparedness. Compared to heterosexual, cisgender women (both White/Caucasian and racial minority) healthcare professionals and heterosexual, cisgender, racial minority men healthcare professionals, healthcare professionals who identified as heterosexual, cisgender, White/Caucasian men reported significantly higher Clinical Preparedness, despite not reporting any other higher LGBT-DOCSS scores. Additionally, many groups reported significantly higher Attitudinal Awareness than heterosexual, cisgender men (both White/Caucasian and racial minority) healthcare professionals. While heterosexual, cisgender, racial minority women reported higher Attitudinal Awareness than heterosexual, cisgender men (both White/Caucasian and racial minority) healthcare professionals, they did not report any other higher LGBT-DOCSS scores. In general, cisgender, sexual minority men and women (both White/Caucasian and racial minority) healthcare professionals reported higher LGBT-DOCSS scores than cisgender, heterosexual men and women (both White/Caucasian and racial minority) healthcare professionals. Gender minority healthcare professionals, in particular those who were also sexual minority healthcare professionals or sexual and racial minority healthcare professionals, reported higher Overall LGBT-DOCSS and Clinical Preparedness than cisgender, heterosexual men and women (both White/Caucasian and racial minority) healthcare professionals.

In all comparisons, similar results were found (i.e., majority of the same significant comparisons remained) after adding total LGBT hours (i.e., both curricular and extracurricular education) as an additional independent variable.

## Discussion

To our knowledge, this study represents the first comprehensive evaluation of LGBT cultural competency among healthcare professionals with multiple minority identities. As the healthcare system is becoming increasingly diverse and collaborative, there has been a shift of focus on the importance of delivering culturally competent care to the LGBT patient population [9]. A systematic review conducted by Sekoni and colleagues [15] supported training of healthcare providers to enhance skills, knowledge, and cultural confidence to provide more equitable healthcare access to LGBT persons. Additionally, recent studies have explored the LGBT competencies of healthcare professionals. Those evaluations found demographic variables to be significant predictors of cultural competency, but the variables (i.e., gender identity, sexual orientation, and race) had not been directly compared [8–10].

The goals of this study were to characterize healthcare professionals’ LGBT cultural competency among demographically diverse groups. It was hypothesized that healthcare professionals with more minority identities would report higher LGBT cultural competency. Compared to men healthcare professionals, women healthcare professionals indicated higher overall competency, yet contrastingly felt less prepared to treat LGBT patients. Perhaps unconscious biases related to self-report could have contributed to women healthcare professionals feeling less prepared, in addition to women’s heightened awareness of gender inequity and feeling that institutions are not meeting diversity goals. For example, Richter et al. [16] conducted a review of a 35-year period and found that compared to men physicians, women physicians in academic medical centers were less likely to be promoted to associate professor, full professor, and/or department chair. Additionally, in the healthcare workplace, despite having similar career aspirations as male counterparts, female faculty are less likely to feel a sense of belonging, feel more aware of gender inequity, and less likely to believe their institutions address diversity goals with faculty positions [16, 17]. As noted above, traditionally, cisgender men have occupied many, if not majority of, leadership roles in academic and healthcare settings. This inequity may contribute to internalized self-doubt and perceived lack of competence in women compared to their men counterparts, which may explain some of the findings in this study. However, more studies are necessary to evaluate how these experiences of gender inequity relate to feeling less prepared to deliver culturally competent care.

Due to their own experiences with discrimination of multiple minority identities, we hypothesized that minority healthcare professionals would display greater LGBT cultural competency. Compared to cisgender, heterosexual healthcare professionals, both sexual minority healthcare professionals and gender minority healthcare professionals reported higher scores. Likewise, previous studies have shown that sexual minority healthcare professionals and gender minority healthcare healthcare professionals often report higher self-assessed comfort, attitudes, and knowledge in caring for LGBT patients than their cisgender, heterosexual peers [10, 18–21]. Higher LGBT cultural competency among sexual minority healthcare professionals and gender minority healthcare professionals is likely a result of more recognition and appreciation for LGBT healthcare and subsequent attainment of advanced education and training secondary to personal identification, values, and experiences with stigma and discrimination. Indeed, LGBT healthcare professionals frequently report enduring bias and hostility from their non-LGBT peers and institutions [6, 21, 22]. At the same time, an investment of congruous LGBT healthcare professionals to provide culturally competent care to patients of their own minority community presents an opportunity to better educate non-LGBT healthcare professionals. Given past evidence that contact with LGBT people leads to improved cultural competency, promotion of safe, welcoming spaces for LGBT healthcare professionals to increase LGBT visibility benefits non-LGBT healthcare professionals as well [23]. However, this opportune teaching and collaboration is often thwarted as many LGBT healthcare professionals fear disclosing their sexual identities and gender identities and perceive low levels of social support from their peers and within institutions [5, 6, 21, 22]. To improve communication and relationships between LGBT and non-LGBT healthcare professionals, more research is needed to understand the lived experiences (i.e., the strengths and barriers) of LGBT healthcare professionals.

Interestingly, we found that White/Caucasian healthcare professionals reported some of the highest LGBT cultural competency scores. Additionally, heterosexual, cisgender, White/Caucasian women healthcare professionals had higher scores than heterosexual, cisgender, racial minority women healthcare professionals. These findings are also somewhat in contrast to data by Smith and Shin [12] that suggested cisgender, heterosexual people of color were able to empathize with the LGBT population based on their own experiences as a minority. On the other hand, similar to data presented here, compared to college-age White women, African American women have reported more negative attitudes towards lesbians and gay men, perhaps due to a relationship between sociocultural influences on attitudes [24]. Culture among White/Caucasian people, with its increasingly accepted gay subculture, resources to combat heterosexism, and influence of feminism, may all facilitate greater acceptance of the LGBT community. Although the White/Caucasian LGBT community continues to face significant sexual prejudice, this community may experience more notable privileges and exposures compared to minority communities because of a widespread mainstream gay culture [24]. Additionally, integrated threat theory proposes that groups may experience a real threat when they perceive another out-group as influencing its existence, political, and/or economic power [24]. Compared to White/Caucasian people, racial minority people often grow and develop within vastly different cultural backgrounds, which may fortify and pose challenges to the perceptions of other groups. Utilizing this conceptualization may explain lower LGBT cultural competency scores among racial minority professionals, such as Black/African American and Asian/Asian American professionals, compared to White/Caucasian professionals. Another consideration for this seeming discrepancy could be related to inherent privilege. For White/Caucasian people, higher income and education may confer increased privilege and decreased discrimination. On the contrary, Black/African American people often experience less privilege and more discrimination with increasing income and education levels [25]. Racial minority providers could also be more aware of discrimination and thereby more attuned to recognizing their own biases.

Interestingly, there were similar comparisons when including the amount of LGBT education as a variable, and separate analyses showed that there were no significant differences in LGBT education across gender identity nor race. Despite similar amounts of LGBT hours received, there may exist group differences in perception, recognition, and appreciation of minority education. Moreover, inherent privilege may confer some, but not all, aspects of cultural competency. For example, White/Caucasian men in particular, presumably with more privilege and resources (but not necessarily more education), reported higher perceived preparedness but much lower positive attitudes and knowledge. Certain cultural competency domains, especially perceived clinical preparedness, are likely more impacted by gender, sexual, and/or racial inequities. These domain-equity differences warrant further exploration in future studies.

## Limitations

There are important study limitations to note. Previous data was collected via convenience samplings, and LGBT cultural competency was assessed via self-report. Consequently, LGBT cultural competency scores may have been inflated, and certain groups may have been more likely to overestimate their competency. Additionally, while thousands of healthcare professionals were polled, they nonetheless represent subgroups of the vast field of healthcare. As such, these respondents and the data herein may not be generalizable to all healthcare professionals. Additionally, although historically underrepresented gender, sexual, and racial minority healthcare professionals were examined, some of these groups were limited by their small sample sizes and consequently may not represent their respective communities. Smaller sample sizes also limited further subgrouping particular professionals (e.g., transgender men, transgender women, nonbinary, specific racial, and multiracial healthcare professionals). Likewise, there may be significant variability in groups, such as intra-group stigma and discrimination; future larger studies should explore intra-group differences in LGBT cultural competency.

## Conclusions

This study represents the first known assessment of LGBT cultural competency among healthcare professionals with multiple minority identities. Compared to their respective counterparts, women healthcare professionals, sexual minority healthcare professionals and gender minority healthcare professionals, and White/Caucasian healthcare professionals reported higher LGBT cultural competency. Given these differences in LGBT cultural competency across intersectionalities of gender identity, sexual orientation, and race among healthcare professionals, future efforts should consider addressing these differences specifically when designing curricula and enhancing available educational services. In doing so, appreciation of strengths and acknowledgment of shortcomings among healthcare professionals will likely result in reductions of biases, tailored education, and thereby translation into improved delivery of appropriate treatment and services for LGBT patients.
